# First Detection of *Leishmania tropica* DNA and *Trypanosoma* Species in *Sergentomyia* Sand Flies (Diptera: Psychodidae) from an Outbreak Area of Cutaneous Leishmaniasis in Ghana

**DOI:** 10.1371/journal.pntd.0002630

**Published:** 2014-02-06

**Authors:** Chukwunonso O. Nzelu, Hirotomo Kato, Naiki Puplampu, Kwame Desewu, Shirley Odoom, Michael D. Wilson, Tatsuya Sakurai, Ken Katakura, Daniel A. Boakye

**Affiliations:** 1 Laboratory of Parasitology, Department of Disease Control, Graduate School of Veterinary Medicine, Hokkaido University, Sapporo, Japan; 2 Department of Parasitology, Noguchi Memorial Institute for Medical Research, University of Ghana, Legon-Accra, Ghana; 3 Ghana Health Service, Accra, Ghana; University of Notre Dame, United States of America

## Abstract

**Background:**

*Leishmania major* and an uncharacterized species have been reported from human patients in a cutaneous leishmaniasis (CL) outbreak area in Ghana. Reports from the area indicate the presence of anthropophilic *Sergentomyia* species that were found with *Leishmania* DNA.

**Methodology/Principal Findings:**

In this study, we analyzed the *Leishmania* DNA positive sand fly pools by PCR-RFLP and ITS1 gene sequencing. The trypanosome was determined using the SSU rRNA gene sequence. We observed DNA of *L. major*, *L. tropica* and *Trypanosoma* species to be associated with the sand fly infections. This study provides the first detection of *L. tropica* DNA and *Trypanosoma* species as well as the confirmation of *L. major* DNA within *Sergentomyia* sand flies in Ghana and suggests that *S. ingrami* and *S. hamoni* are possible vectors of CL in the study area.

**Conclusions/Significance:**

The detection of *L. tropica* DNA in this CL focus is a novel finding in Ghana as well as West Africa. In addition, the unexpected infection of *Trypanosoma* DNA within *S. africana africana* indicates that more attention is necessary when identifying parasitic organisms by PCR within sand fly vectors in Ghana and other areas where leishmaniasis is endemic.

## Author Summary

Cutaneous leishmaniasis (CL) is one of the world's most neglected diseases transmitted by female sand flies and affecting mostly developing countries with about 1.2 million cases every year. In most African countries, the disease is typically caused by one of two species of *Leishmania* parasite: *L. major* or *L. tropica*. Clinical symptoms of both infections are similar, producing ulcerative and nodular lesions. Notwithstanding their similarity, lesions caused by *L. major* self-heal and bestow immunity to re-infection and therapy, if applied, is often by antimonials administered intra-lesionally, whereas the treatment of CL caused by *L. tropica* is difficult. Differentiating between these agents in any CL focus is important. Following the outbreak of CL in Ghana, we focused on the sand fly species present in the area in order to detect *Leishmania* DNA in potential vectors. Our study provides evidence on the occurrence of *L. tropica* and *L. major* DNA, and the detection of *Trypanosoma* DNA in *Sergentomyia* sand flies in Ghana. These findings have considerable implications in determining the epidemiology and dynamics of the disease. Significantly, our study supports the possibility of *Sergentomyia* sand flies as the vectors of CL in Ghana other than *Phlebotomus*, which contains all currently known vectors for *Leishmania* in the Old World.

## Introduction

Cutaneous leishmaniasis (CL) is a vector-borne parasitic disease of humans and other mammals caused by cell-infecting flagellate protozoa of the genus *Leishmania*, transmitted by female phlebotomine sand flies. The disease occurs in 98 tropical, subtropical and temperate countries worldwide and it is estimated that 1.2 million new cases of CL occur per year [1]. CL of the Old World is caused by five species of *Leishmania*: *L. tropica*, *L. major*, *L. aethiopica*, *L. infantum* and *L. donovani* [2]. Clinically, cutaneous lesions due to *L. tropica* last much longer and are more difficult to treat than those due to *L. major* [3]. To date, there is no information available on the distribution of *L. tropica* in West Africa.

Sand fly species of the genus *Phlebotomus* are the putative vectors of *Leishmania* in the Old World [4]; however, few studies have recently suggested the possible involvement of some species of the genus *Sergentomyia* in the transmission of *Leishmania* in the Old World. Studies conducted in cutaneous leishmaniasis foci in Iran, Mali and Portugal have shown the detection of *L. major* DNA in *S. sintoni* [5], *S. darlingi* [6] and *S. minuta* [7], respectively. Earlier, *L. major* was also isolated from *S. garnhami* in Kenya [8]. Other reports have also detected *L. donovani* DNA in *S. babu* in India [9] and more recently, *L. siamensis* DNA in *S. gemmea* in Thailand [10].

In Ghana, *L. major* [11] and uncharacterized species [12] have been identified as the causative agents of human cutaneous leishmaniasis in an area of CL outbreak. In order to control the transmission of *Leishmania* spp. in an endemic area, information on potential sand fly vectors as well as their associated *Leishmania* species is of paramount importance since vector dispersion is one of the major factors that determine the potential rate of pathogen dissemination. Thus, this study was designed to determine and detect *Leishmania* DNA within sand flies collected in the outbreak area of CL in Ghana by molecular analysis in order to ascertain if they were infected by either *L. major* [11] or the yet to be characterized species [12]. This information is necessary to implicate the potential vectors in the area for a better understanding of the epidemiology of leishmaniasis in Ghana. Here, we report the confirmation of *L. major* DNA and first detection of *L. tropica* DNA in *Sergentomyia* species using polymerase chain reaction-restriction fragment length polymorphism (PCR-RFLP) and DNA sequencing analyses, as well as *Trypanosoma* species associated with sand fly infections in Ghana.

## Materials and Methods

### Sand fly capture and taxonomic identification

Previously collected indoor resting sand flies from three CL outbreak communities; Klefe, Hlefi and Taviefe in Ho District of Ghana were used in this study. Verbal informed consent was obtained from residents in each of the community. The collections were done in August, September and November 2007 from human habitats using manual aspirator. Each unfed and blood-fed female fly was dissected under sterile conditions by cutting off the head and last three abdominal segments with a pair of sterilized entomological needles and forceps. The head and last three abdominal segments of each fly was mounted on microscope slides in Puri's medium and identified using taxonomic keys [13], [14]. For each blood-fed fly, the remainder of the body was processed under sterile condition and subjected to blood-meal analysis (Boakye et al, unpublished results). Afterwards, the dissected thorax and attached anterior abdomen of unfed and the processed specimens known to have fed on human-blood (Boakye et al, unpublished results) were separately pooled according to species and locality (Table 1) for DNA extraction and infection molecular analysis.

**Table 1:** Summary of *Sergentomyia* sand flies screened by molecular biological method in this study.

Location	Sand fly species	Number of unfed pools* (positive pools)	Number of blood-fed pools* ¶ (positive pools)	Total
**Klefe**	*S. africana africana*	5	1	6
	*S. ingrami*	10	2 (1)^c^	12
	*S. simillima*	12	2	14
	*S. dissimillima*	13	0	13
	*S. hamoni*	5 (1)^a^	0	5
	**Total**	**45**	**5**	**50**
**Hlefi**	*S. africana africana*	19 (1)^b^	1	20
	*S. ingrami*	7 (2)^c^	1	8
	*S. simillima*	4	2	6
	*S. dissimillima*	5	0	5
	**Total**	**35**	**4**	**39**
**Taviefe**	*S. africana africana*	4	0	4
	*S. ingrami*	4 (1)^a^	1	5
	*S. simillima*	3	0	3
	*S. dissimillima*	1	0	1
	**Total**	**12**	**1**	**13**

* Ten female specimens in each pool.

¶ Identified human-blood fed specimens (Boakye et al, unpublished results).

a *L. tropica* DNA detection according to sequence analysis.

b *Trypanosoma* sp. DNA detection according to sequence analysis.

c *L. major* DNA detection according to sequence analysis.

### DNA extraction, *Leishmania* ITS1 PCR-RFLP and trypanosomatid SSU rRNA gene amplification

Ten (10) unfed and blood-fed flies belonging to the same species were pooled and homogenized in animal tissue lysis buffer (Qiagen) containing proteinase K. DNA was extracted using the Qiagen DNA mini kit (Qiagen, Valencia, CA) according to the manufacturer instructions and 0.5 µl of the extract was used as PCR templates.

The leishmanial ribosomal internal transcribed spacer 1 (ITS1) region was amplified, using primers L5.8S and LITSR [15]. The reactions were carried out in volumes of 25 µl containing 200 µM of each dNTP, 1.5 mM MgCl_2_, 2 units Taq polymerase and 500 nM of each primer. Each PCR reaction included a positive control (DNA from a reference strain: *L. major* - IPAP/EG/1989/S1-177 and *L. tropica* - MHOM/SU/1974/K27) and a negative control (water). After initial denaturation at 95°C for 2 min, PCR amplification were performed with 34 cycles consisting of denaturation (95°C for 20 sec), annealing (53°C for 30 sec), and extension (72°C for 1 min) followed by a final extension cycle at 72°C for 6 min. An aliquot of 5 µl of each PCR reaction was separated by electrophoresis on 2% agarose gel and visualized. Each ITS1 amplicon was digested with the restriction endonuclease *Hae*III (Invitrogen) for the species identification. The restriction fragments were run and visualised on 2% agarose gel and compared with those of reference strains of *L. major* (IPAP/EG/1989/S1-177) and *L. tropica* (MHOM/SU/1974/K27).

For the identification of *Trypanosoma* species, the small subunit ribosomal RNA (SSU rRNA) gene was amplified from the sand fly using primer specific for SSU rRNA gene of trypanosomatids (TRY927F: GAAACAAGAAACACGGGAG and TRY927R: CTACTGGGCAGCTTGGA) [16], [17], [18].

### PCR amplification of sand fly COI and18S rRNA genes

Validation of morphologically identified sand fly specimens was done using mitochondrial cytochrome c oxidase gene subunit I (COI) and 18S rRNA genes. Amplification of the COI gene was accomplished using the primers LCO I490 (5′-GGTCAACAAATCATAAAGATATTGG3′) and HCO 2198 (5′TAAACTTCAGGGTGACCAAAAAATCA-3′) [19]. The reaction was carried out in a volume of 15 µl using a pair of primers (1 µM each) and 2× AmpliTaq Gold PCR Master Mix (Applied Biosystems, NJ, USA). After initial denaturation at 95°C for 5 min, amplification was performed with 37 cycles consisting of denaturation at 94°C for 30 sec, annealing at 55°C for 45 sec, extension at 72°C for 1 min 30 sec, followed by a final extension at 72°C for 10 min. For the 18S rRNA locus, the sequence of interest was amplified using the primers Lu. 18S rRNA-1S (5′-TGCCAGTAGTTATATGCTTG-3′) and Lu. 18S rRNA-1R (5′-TTACGCGCCTGCTGCCTTCC-3′) [20]. The reaction was carried out in a volume of 15 µl using a pair of primers (0.4 µM each) and 2× AmpliTaq Gold PCR Master Mix (Applied Biosystems, NJ, USA). An initial denaturation was done for 5 min at 95°C, followed by PCR amplification for 30 cycles of denaturation (95°C for 1 min), annealing (55°C for 1 min), and polymerization (72°C for 1 min), with a final extension of 10 min at 72°C. Amplified products were resolved on 2% agarose gels.

### Molecular cloning and nucleotide sequencing

Infection and sand fly PCR products were directly cloned into plasmid using a pGEM-T Easy Vector System (Promega, Madison, WI). *Escherichia coli* DH5 α cells was transformed with a ligated product and plated onto Luria Bertani agar plates containing ampicillin (50 µg/ml), 5-bromo-4-chloro-3-indolyl β-D-galactoside (36 µg/ml), and isopropyl β-D-thiogalactoside (40 µg/ml). Plasmid DNAs were extracted with QIAprep Spin Miniprep Kit (Qiagen) and the insert of the plasmids were sequenced by the dideoxy chain termination method using BigDye Terminator version 3.1 Cycle Sequencing Kit (Applied Biosystems, Foster City, CA).

### Phylogenetic analysis

Sequences from both strands were aligned using Clustal W software [21] and imported into MEGA (Molecular Evolutionary Genetics Analysis) version 4.0 [22]. Phylogenetic trees were constructed by the neighbor-joining (NJ) method with the algorithm of MEGA program. Bootstrap values were determined with 1,000 replicates of the datasets. ITS1 gene sequences used for the analysis were *L. major* (IPAP/EG/1989/SI-177, DQ295824; MHOM/GH/2004/HO-004, DQ295825; MHOM/EG/2006/RTC-64, FJ460456; MHOM/TN/1997/LPN162, FN677342; MHOM/IL/1967/JERICHO II, EU326229), *L. tropica* (MHOM/EG/2006/RTC-66, FJ460457; IROS/NA/1976/ROSSI-II, AJ000302; MHOM/KE/1984/NLB297, AJ000301; MHOM/TN/1988/TAT3, AJ300485) and *Leishmania* sp. (MHOM/GH/2006/TVE, EF524071). The trypanosomatid SSU rRNA gene sequence was analyzed with those of *T. avium* (AB566384, AF416559), *T. brucei rhodesiense* (AJ009142), *T. corvi* (AY461665), *T. microti* (AJ009158), *T. kuseli* (AB175626), *T. cruzi* (AJ009149, AJ009150), *T. rangeli* (AJ012417), *T. congolense* (AJ009145, AJ009146), *T. mega* (AJ009157), *T. rotatorium* (AJ009161), *T. fallisi* (AF119806) and *Trypanosoma* species isolated from toads (EU021231, EU021232), *Lutzomyia* sand flies (EU021241, EU021242, EU021243, EU021244, EU021245, EU021237), *Phlebotomus* sand fly (AB520638), mosquitoes (AF416561), and hippoboscid (AF416562).

Sand fly COI gene sequences were analyzed with those of *Sergentomyia babu babu* (HQ585351, HQ585357), *S. vadhanurensis* (HQ585345, HQ585348), *S. bailyi* (HQ585381 and HQ585384), *S. punjabenensis* (HQ585375, HQ585379), *Phlebotomus papatasi* (JN172077) and the New World sand fly species *Lutzomyia longiflocosa* (FJ437273). The sand fly 18S rRNA gene sequences were analyzed with those of *S. barraudi* (JQ790518), *S. queenslandi* (HM775498), *S. magna* (AJ391741), *S. babu* (JN581685), *S. minuta* (AJ244419), *S. schwetzi* (AJ391739), *S. buxtoni* (AJ391737), *S. dentata* (AJ244423), *S. dubia* (AJ391738), *S. clydei* (AJ391742), *S. ghesquierei* (AJ391743), *P. papatasi* (AJ391726) and the New World sand fly species *Lu. longipalpis* (AJ244429).

### Statistical analysis

The infection rate in sand flies was determined using the poolScreen2 program generously provided by Dr. Charles Katholi (Department of Biostatistics and Division of Geographic Medicine, University of Alabama at Birmingham, USA) [23]. The algorithm was used to calculate the maximum likelihood estimate (MLE) of infection in sand fly species at 95% confidence intervals.

### Accession numbers

AB759711 508df2892113a5dbfe0001d5.clone1

AB759712 508df2892113a5dbfe0001d5.clone2

AB759713 508df2892113a5dbfe0001d5.clone3

AB759714 508f999a2113a5dfe60001a2.cloneIS1

AB759715 508f999a2113a5dfe60001a2.cloneIS2

AB759716 508f999a2113a5dfe60001a2.cloneIS3

AB759971 508fa9992113a5dbfe00028e.cloneCOI1

AB759972 508fa9992113a5dbfe00028e.cloneCOI2

AB759973 508fa9992113a5dbfe00028e.cloneCOI3

AB787189 511af92b12c2e8dff9003639.clone4

AB787190 511af92b12c2e8dff9003639.clone5

AB787191 511af92b12c2e8dff9003639.clone6

AB787192 511af92b12c2e8dff9003639.cloneCOI4

AB787193 511af92b12c2e8dff9003639.cloneCOI5

AB787194 511af92b12c2e8dff9003639.cloneCOI6

AB787195 511af92b12c2e8dff9003639.cloneIS4

AB787196 511af92b12c2e8dff9003639.cloneIS5

AB787197 511af92b12c2e8dff9003639.cloneIS6

## Results

### Detection of trypanosomatids within sand flies

**Figure 1:**
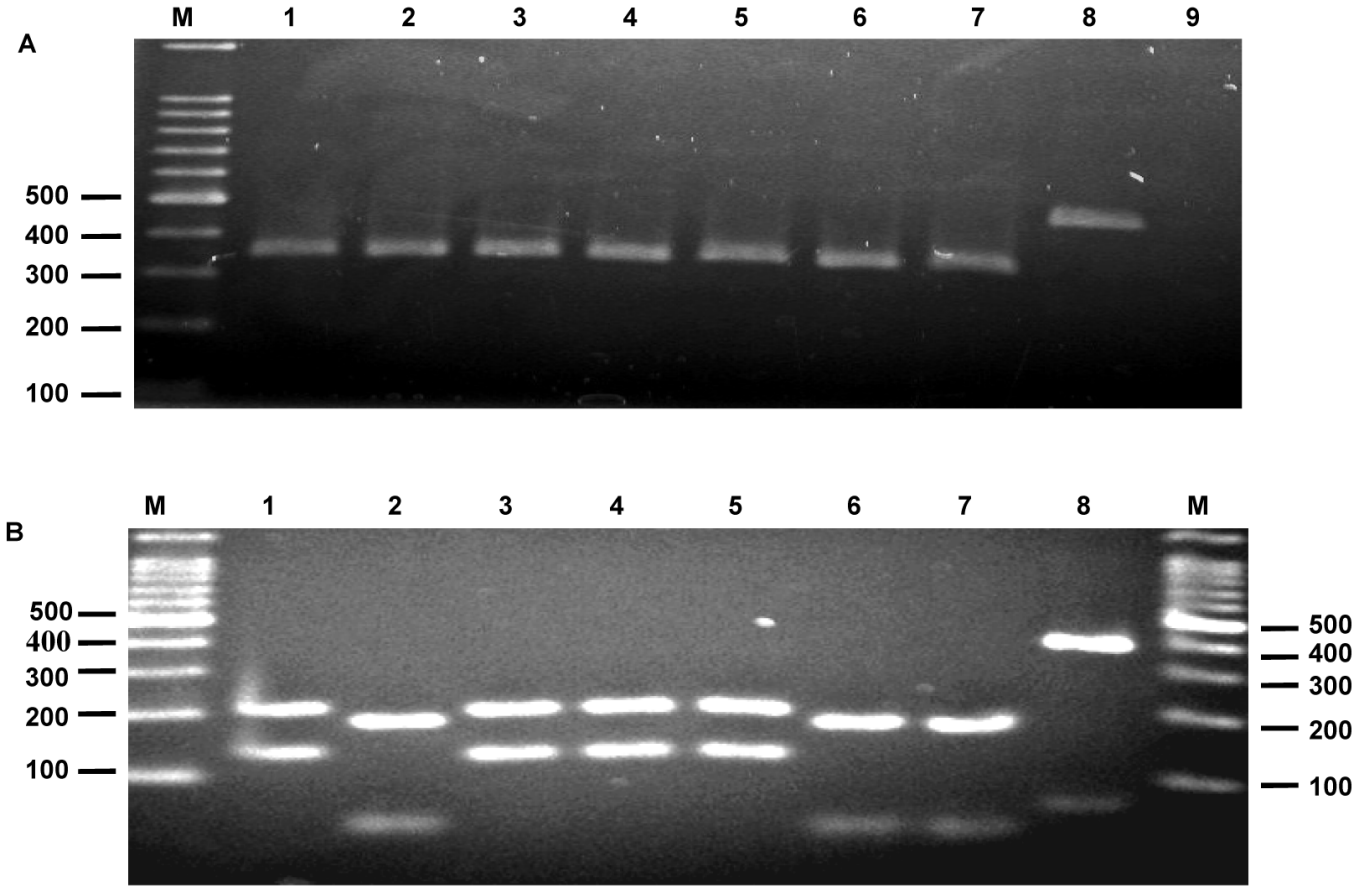
Detection of *Leishmania* DNA in sand flies. **A.** PCR of *Leishmania* internal transcribed spacer 1 (ITS1) region amplified within pools of female *Sergentomyia* sand flies captured indoors and *Leishmania* spp. controls. M: 100 bp size marker; Lanes 1 and 2 (*L. major* and *L. tropica* reference strains, respectively); Lanes 3 to 6, *S. ingrami* pools, Lane 7, *S. hamoni* pool; Lane 8, *S. africana africana* pool (∼500 bp) and Lane 9, negative control. **B.** *Hae*III digestion of restriction fragment length polymorphisms of ITS1 PCR products shown in A. M: 100 bp size marker; Lane 1, *L. major* (IPAP/EG/1989/S1-177); Lane 2, *L. tropica* (MHOM/SU/1974/K27); Lanes 3 to 6, *S. ingrami* pools; Lane 7, *S. hamoni* pool and Lane 8, *S. africana africana* pool.

**Figure 2:**
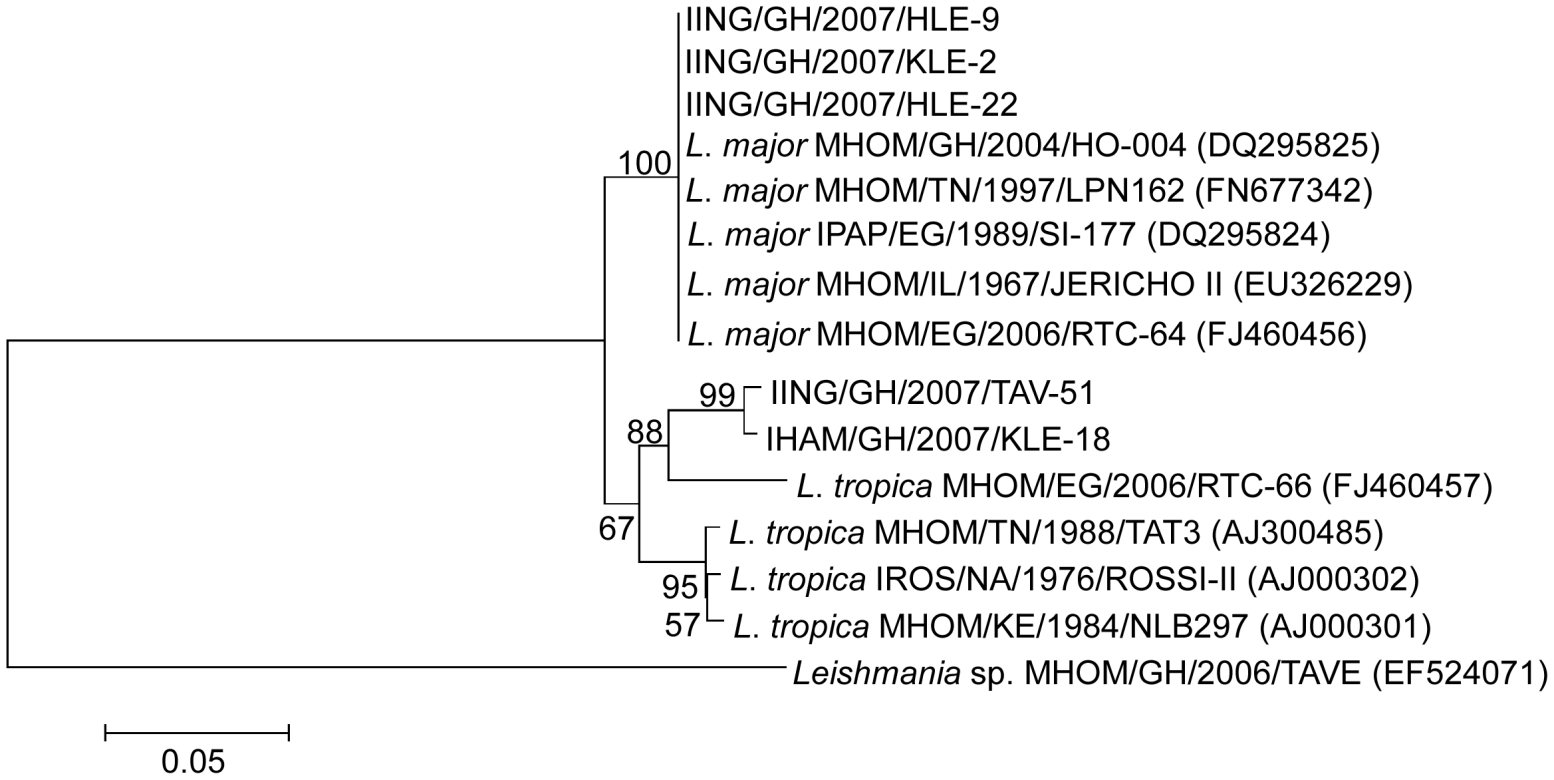
Phylogenetic tree of ITS1 gene sequences among species. *Leishmania* ITS1 gene was amplified within *Leishmania* DNA positive *S. ingrami* pools (IING/GH/2007/HLE-9, IING/GH/2007/KLE-2, IING/GH/2007/HLE-22 and IING/GH/2007/TAV-51) and *S. hamoni* pool (IHAM/GH/2007/KLE-18) and sequenced. Analysis was performed by the neighbor-joining method on the sequences together with those from 10 *Leishmania* species including the 2 human isolates from Ghana, *L. major* (MHOM/GH/2004/HO-004) and an uncharacterized *Leishmania* sp. (MHOM/GH/2006/TAVE). The scale bar represents 0.05% divergence. Bootstrap values are reported at nodes.

*Sergentomyia* sand flies of 102 pools were analyzed (Table 1), each containing ten females (in total 1,020 insects): *S. africana africana* (2 blood-fed, 28 unfed pools), *S. ingrami* (4 blood-fed, 21 unfed pools), *S. simillima* (4 blood-fed, 19 unfed pools), *S. dissimillima* (19 unfed pools), and *S. hamoni* (5 unfed pools). ITS1-PCR showed 6 positives, with a unique band of approximately 340 bp in 5 pools, namely, 4 *S. ingrami* and 1 *S. hamoni* as expected from the results obtained from the reference strains and the other yielding a fragment of approximately 500 bp in 1 *S. africana africana* pool (Fig. 1A). No *Leishmania* DNA was detected in *S. simillima* and *S. dismillima* pools from the three CL foci. The six positive samples were subjected to RFLP for species identification and their subsequent digestion with the endonuclease *Hae*III produced fragments characteristic of *L. major* in samples 3–5 (reported in: Boakye et al, unpublished results) and the *L. major* reference DNA, and *L. tropica* in samples 6–7 and the *L. tropica* reference DNA, whereas sample 8 displayed an uncharacteristic RFLP pattern (Fig. 1B). To confirm authenticity of the RFLP analysis, both the 340 bp and 500 bp ITS1 PCR products obtained in the sand fly positive samples were subjected to sequencing analysis. The ITS1 DNA sequences obtained from the 3 (1 blood-fed from Klefe, 2 unfed from Hlefi) *S. ingrami* pools [IING/GH/2007/HLE-9 (GeneBank accession number: AB759711), IING/GH/2007/KLE-2 (AB759712), and IING/GH/2007/HLE-22 (AB759713)] were identical to each other and showed 99% identity to several *L. major* ITS1 sequences in GenBank, including isolates from Ghana, Egypt, Kenya, Sudan and Tunisia. The ITS1 sequences obtained from 1 unfed *S. ingrami* pool from Taviefe [IING/GH/2007/TAV-51 (AB787189)] and 1 unfed *S. hamoni* pool from Klefe [IHAM/GH/2007/KLE-18 (AB787190)] were different at 4 positions of the nucleotides, which include the deletion of 2 nucleotides in the former. These samples showed high similarity with several *L. tropica* ITS1 sequences in GenBank at 99% identity. In the phylogenetic tree, the 3 *L. major* sequences determined in this study were grouped into *L. major* cluster with 100% bootstrap support value, and the 2 *L. tropica* sequences determined were located in *L. tropica* cluster (Fig. 2).

**Figure 3:**
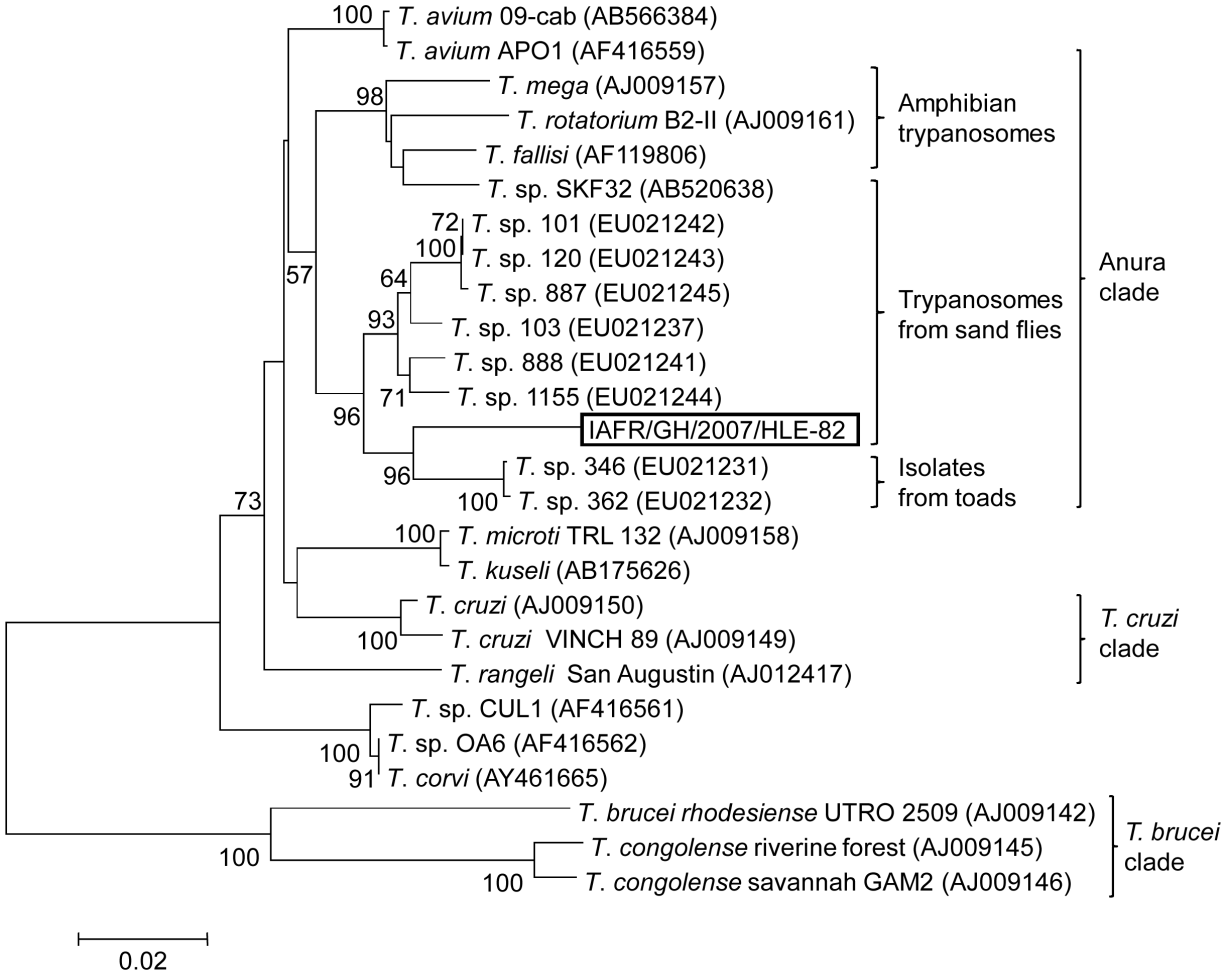
Phylogenetic tree of SSU rRNA gene sequences among species. The SSU rRNA (IAFR/GH/2007/HLE-82) gene was amplified within *S. africana africana* pool and sequenced. Phylogenetic analysis of the SSU rRNA gene sequences was performed by the neighbor-joining method on the sequence together with those from 25 *Trypanosoma* species. The sequences from the database are represented by the name of the species, isolates and GeneBank (accession number). The scale bar represents 0.02% divergence. Bootstrap values are reported at nodes.

The ITS1 gene sequence of the 500 bp amplicon was determined and analyzed with the NCBI BLAST. Unexpectedly, the sequence did not match with those of *Leishmania* species but rather showed similarity to *Trypanosoma* spp., suggesting that the DNA sequence obtained belongs to the genus *Trypanosoma*. For further identification, the sample was analyzed with the SSU rRNA gene because this gene has been well studied in trypanosomatids [16], [17], [18]. The SSU rRNA gene sequence obtained [IAFR/GH/2007/HLE-82 (AB787191)] had 93% identity with those of trypanosomes isolated from toads. A phylogenetic tree constructed with this sequence and SSU rRNA sequences of *Trypanosoma* species showed that the *Trypanosoma* DNA sequence determined from *S. africana africana* pool collected from Hlefi was located in the clade comprising trypanosomes isolated from Brazilian toads (*Trypanosoma* sp. 362 and 364) [24], but the sequence did not completely match those from any reported species (Fig. 3). In addition, the *Trypanosoma* SSU rRNA sequence determined was separated from *Trypanosoma* species isolated from Amazon sand flies (*T.* sp. 101, 120, 887, 103, 888, and 1155) [24] and in a desert area of Pakistan (*T.* sp. SKF32) [18], which have closer relationships with anuran trypanosomes (Fig. 3). This result suggests that the *Trypanosoma* DNA detected within *S. africana africana* in this study is a novel or genetically uncharacterized *Trypanosoma* species possibly of amphibians.

The minimum infection rate, assuming that one infected insect was present in each positive pool was 0.49% for *Leishmania* spp. and 0.10% for *Trypanosoma* infection among the total population of pooled female sand flies. Using poolScreen2 algorithm, the maximum *Leishmania* DNA infection rate was estimated as follows: *L. tropica* infection rate in *S. ingrami* (1 out of 25 pools) and *S. hamoni* (1 out of 5 pools) were 4.07% (95% CI 1.259–2.080) and 2.21% (95% CI 6.864–1.094), respectively. *Trypanosoma* DNA infection in *S. africana africana* (1 out of 30 pools) was estimated at 3.38% (95% CI 1.045–1.731).

### Validation of sand fly species by molecular biological method

**Figure 4:**
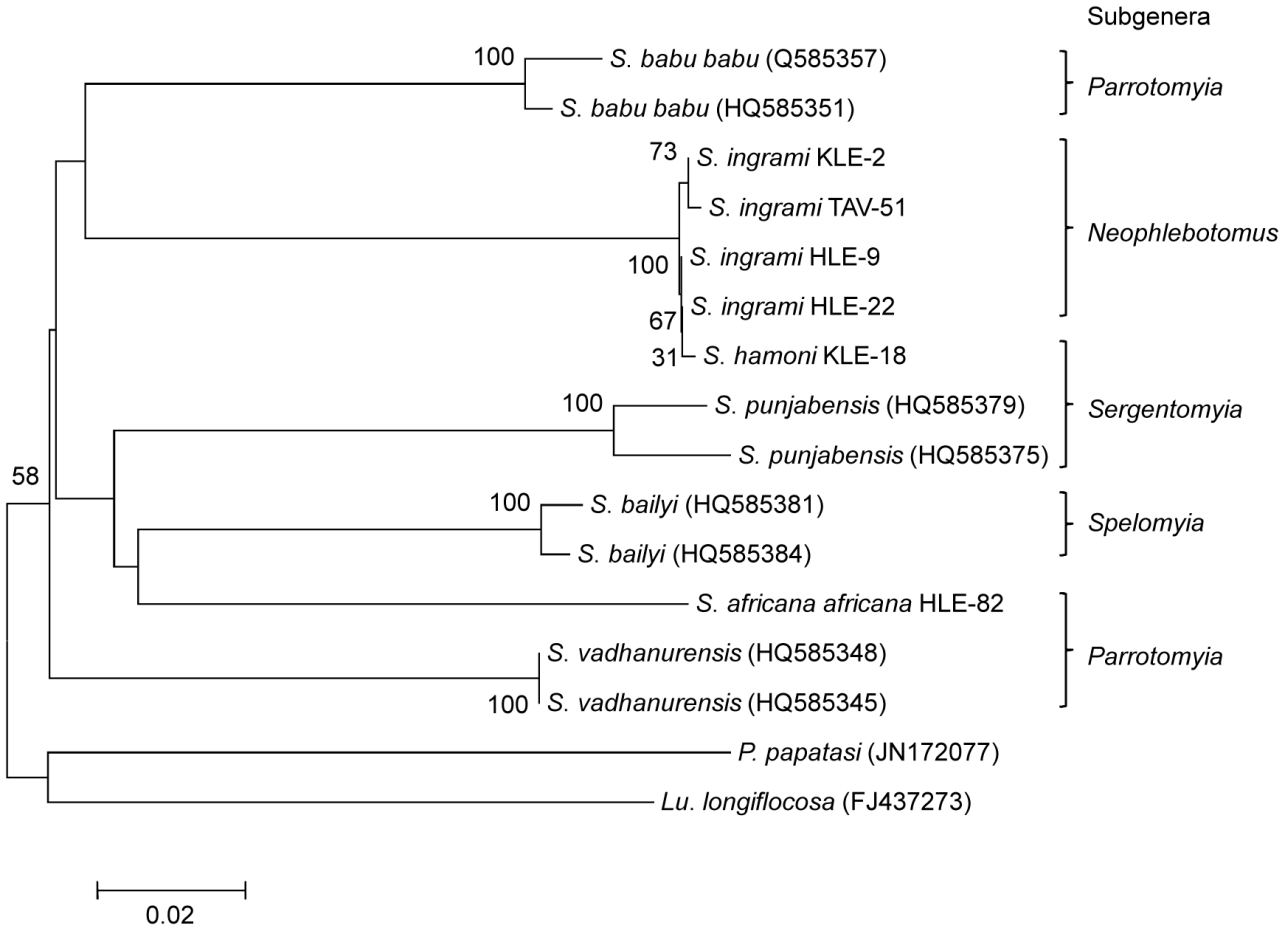
Phylogenetic tree of COI gene sequences among sand fly species. The COI (*S. ingrami* HLE-9, *S. ingrami* HLE-22, *S. ingrami* KLE-2, *S. ingrami* TAV-51, *S. hamoni* KLE-18 and S. *africana africana* HLE-82) gene was amplified from infected sand flies and sequenced. Analysis of the sequences together with those from *Sergentomyia* species, *Phlebotomus* species and *Lutzomyia* species registered in GeneBank were performed. The bar scale represents 0.02% divergences. Bootstrap values are shown above or below branches. HLE, Hlefi community; KLE, Klefe community; TAV, Taviefe community.

**Figure 5:**
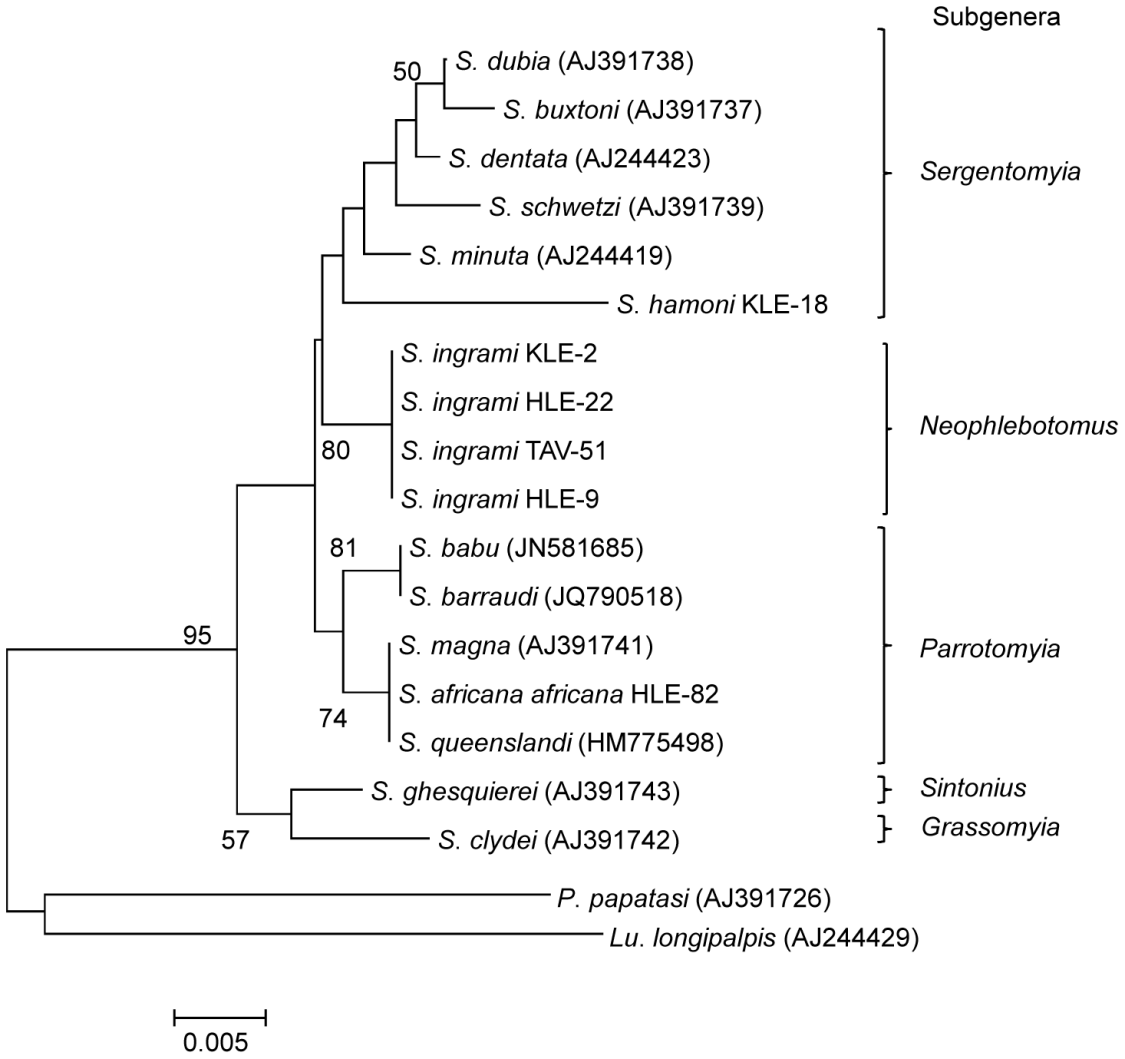
Phylogenetic tree of 18S rRNA gene sequences among sand fly species. The 18S rRNA (*S. ingrami* HLE-9, *S. ingrami* HLE-22, *S. ingrami* KLE-2, *S. ingrami* TAV-51, *S. hamoni* KLE-18 and S. *africana africana* HLE-82) gene was amplified from infected sand flies and sequenced. Analysis of the sequences together with those from *Sergentomyia* species, *Phlebotomus* species and *Lutzomyia* species registered in GeneBank were performed. The bar scale represents 0.005% divergences. Bootstrap values are shown above or below branches.

The COI gene sequences from infected flies (accession numbers: AB759971–AB759973, AB787192–AB787194) showed greater homology with those of *Sergentomyia* species registered in GeneBank than any other genus. A phylogenetic analysis was performed to observe the phylogenetic relationships of COI among *Sergentomyia* species. Among the three different infected *Sergentomyia* species included in phylogenetic analysis, only *S. africana africana* of the subgenus *Parrotomyia* was monophyletic (Fig. 4). All the four *S. ingrami* and one *S. hamoni* samples of the subgenera *Neophlebotomus* and *Sergentomyia*, respectively, were clustered in the same clade (Fig. 4). Similarly, 18S rRNA gene sequences from infected flies (accession numbers: AB759714–AB759716, AB787195–AB787197) had a greater degree of homology with those of *Sergentomyia* species (98–99%) than any *Phlebotomus* and *Lutzomyia* species (92–97%). In the phylogenetic tree, all species characterized in this study and those of other *Sergentomyia* species were located in their respective subgenus cluster (Fig. 5). These results confirmed that sand flies infected by *Leishmania* DNA belong to the genus *Sergentomyia*.

## Discussion

In this study, we report the detection of *Leishmania* DNA within *Sergentomyia* species and their comparison with *Leishmania* species previously isolated from humans in Ho District. The correct identification and comparison of *Leishmania* species from sand flies and humans is of epidemiological importance for predictions of the risk and expansion of the disease in endemic and surrounding areas. Different PCR-based techniques are applied either in individual sand flies [6], [20] or in sand fly pools [25].

Three cutaneous leishmaniasis foci Klefe, Hlefi and Taviefe were identified in Ho, where both *L. major* [11] and uncharacterized species [12] were found as the etiologic agents. In the present study, only sand fly species of the genus *Sergentomyia* were identified from the CL focal area, consistent with previous reports from the same area [26], [27] and suggests the possible involvement of this genus in the transmission of CL. Our study revealed infection of *L. major* DNA in *S. ingrami* pools, and *L. tropica* DNA in one *S. ingrami* and one *S. hamoni* pools by ITS1 PCR-RFLP and sequencing analyses. These observations represent the first detection of *L. tropica* DNA and confirmation of *L. major* DNA in sand flies by gene sequencing in Ghana and support the earlier hypothesis that species of the genus *Sergentomyia* might be the vectors of CL in this area [26], [27]. The detection of *L. tropica* DNA in Ho focus is a novel finding and the first report in West Africa. In addition, DNA of *Trypanosoma* species was detected within one *S. africana africana* pool by the SSU rRNA gene analysis, which also represents the first report on infection of sand fly by trypanosomes other than *Leishmania* in West Africa. Furthermore, the blood-meal analysis of the blood-fed flies used in this study, revealed 3 anthropophilic *Sergentomyia* species; *S. ingrami*, *S. africana africana* and *S. simillima* (Boakye et al, unpublished results), [26].

Vector incrimination is an important part of any epidemiological studies on leishmaniasis. The vector role of a sand fly species in leishmaniasis focus is epidemiologically suspected when the species is predominant and proved anthropophilic behavior. This suspicion is strengthened when the same sand fly is found infected with the same leishmanial parasite as that found in man in the same place [28]. Phylogenetic analysis of the leishmanial ribosomal internal transcribed spacer 1 region indicates that the sequences from *L. major* DNA determined from *S. ingrami* align most closely with a human isolate from Ghana (MHOM/GH/2004/HO-004) [11]. Thus, based on the documented anthropophilic nature of *S. ingrami* (Boakye et al, unpublished results), [26], its abundance and the detection of *L. major* DNA within this sand fly as the same *Leishmania* species found in man from the same CL focus, suggests that *S. ingrami* is a possible vector of *L. major* transmission in the study area.

The amplification of *Leishmania* DNA in *S. ingrami* and *S. hamoni*, collected in Taviefe and Klefe communities, respectively, which clustered together with other ITS1 sequences of *L. tropica*, suggests that *L. tropica* might also be associated with the cases of CL in the outbreak area. Although phylogenetic analysis indicates that the ITS1 sequences from *L. tropica* DNA in Ghana have close affinity to an African isolate of *L. tropica* from Egypt, they were divergent from reported African strains. *L. tropica* is recognized as a very heterogeneous species of *Leishmania* and intra-specific microheterogeneity has been readily demonstrated [29]. The lack of information on the existence of *L. tropica* in human in this focus might be largely due to the small numbers of human specimens investigated [11], [12] and difficulties in distinguishing between *L. major* and *L. tropica*. The emergence of human CL due to *L. tropica* as an increasingly important public health problem is now being reported in many foci in Africa such as Libya, Kenya, Egypt and Morocco [30]–[33]. To our knowledge, this study presents the first detection of *L. tropica* DNA in *Sergentomyia* species.

The detection of *L. tropica* DNA in *S. ingrami* and *S. hamoni* in this area indicates the possibility of the two species participating in the parasite transmission cycle. The conventional wisdom was that there is specificity of *Leishmania* species and the vector hosts. However, it has been demonstrated that most sand fly species can support the development of multiple *Leishmania* species [34]. The mechanism of *Leishmania* attachment in permissive sand fly vectors as seen in *Lutzomyia longipalpis* and *Phlebotomus arabicus* was shown to be independent of midgut lipophosphoglycan (LPG) [35], whether such reported LPG-independent mechanism will apply to all permissive sand flies remains to be determined.

Another important finding is the infection of *Trypanosoma* DNA within *S. africana africana*. Phylogenetic analysis indicates that the SSU rRNA sequence from *Trypanosoma* DNA determined from *S. africana africana* in Ghana has a closer relationship with anuran trypanosomes, but was significantly divergent from all the reported strains [18], [24]. These observations strongly suggest that *S. africana africana* was infected by a novel or an uncharacterized *Trypanosoma* species. Several sand fly species have been reported to transmit *Trypanosoma* species in tropical and subtropical areas and most parasites were anuran trypanosomes [18], [36], [37]. To date, there is no data in the literature about the natural infections of *S. africana africana* by trypanosomes. The result presented in this study reinforces the importance of correct identification of parasitic organisms within insect vectors.

We have characterized for the first time DNA barcodes and 18S rRNA gene of *S. ingrami*, *S. hamoni* and *S. africana africana*. However, the sand fly COI phylogenetic analysis places both *S. ingrami* and *S. hamoni* in the same clade while the 18S rRNA gene tree separates them, several factors may explain species non-monophyly [38], but the most likely explanation, therefore, is incomplete lineage sorting. Notwithstanding this, both genes confirmed that all the infected sand fly species belonged to the genus *Sergentomyia* and support our evidence for species of this genus as the possible vectors of *Leishmania* in Ghana. Our findings question the dogma that *Leishmania* is exclusively transmitted by species of the genus *Phlebotomus* in the Old World. This observation warrants further investigation by microscopical examination of these sand fly species to obtain information on the localization of infection and presence of metacyclics, as well as initiating experimental studies in order to demonstrate their capacity to transmit the parasites. Additionally, surveillance would also be intensified aiming to identify the natural reservoir hosts in order to improve our understanding of the epidemiology of CL in the outbreak area.

Although sand flies of the genus *Sergentomyia* are considered as vectors of reptile *Leishmania* species, non-pathogenic to human [39], and *S. schwetzi* reported to be refractory to human *Leishmania* species [40], our data adds to few reported studies on the detection of human pathogenic *Leishmania* species in some *Sergentomyia* species: *S. sintoni* [5], *S. darlingi* [6], *S. minuta* [7], *S. garnhami* [8], *S. babu* [9] and *S. gemmea* [10], incriminating them as potential vectors of mammalian *Leishmania* species. Definitive conclusion that these *Sergentomyia* species are vectors of human *Leishmania* species awaits confirmation by demonstrating experimentally their capacity to transmit *Leishmania* parasite by biting to mammals. Though it is technically difficult to establish laboratory colonies of some species of sand flies and infection studies therein, it remains highly desirable for vector incrimination.

Taken together, the presence of multiple of *Leishmania* spp. in this CL focus suggests a more complex epidemiology for this outbreak than anticipated. Therefore, a proper understanding of the different parasites' life cycles and parasite-vector-reservoir interplays is needed for predicting the risk and expansion of the disease and applying effective prevention and control strategies in Ghana.
